# Metformin-mediated growth inhibition involves suppression of the IGF-I receptor signalling pathway in human pancreatic cancer cells

**DOI:** 10.1186/1471-2407-13-235

**Published:** 2013-05-10

**Authors:** Emelie Karnevi, Katarzyna Said, Roland Andersson, Ann H Rosendahl

**Affiliations:** 1 Department of Surgery, Clinical Sciences Lund, Skåne University Hospital and Lund University, Lund SE-221 84, Sweden

**Keywords:** Pancreatic cancer, Type 2 diabetes mellitus, Hyperglycaemia, Metformin, Insulin-like growth factor (IGF), Signalling

## Abstract

**Background:**

Epidemiological studies have shown direct associations between type 2 diabetes and obesity, both conditions associated with hyperglycaemia and hyperinsulinemia, and the risk of pancreatic cancer. Up to 80% of pancreatic cancer patients present with either new-onset type 2 diabetes or impaired glucose tolerance at the time of diagnosis. Recent population studies indicate that the incidence of pancreatic cancer is reduced among diabetics taking metformin. In this study, the effects of exposure of pancreatic cancer cells to high glucose levels on their growth and response to metformin were investigated.

**Methods:**

The human pancreatic cancer cell lines AsPC-1, BxPC-3, PANC-1 and MIAPaCa-2 were grown in normal (5 mM) or high (25 mM) glucose conditions, with or without metformin. The influence by metformin on proliferation, apoptosis and the AMPK and IGF-IR signalling pathways were evaluated *in vitro*.

**Results:**

Metformin significantly reduced the proliferation of pancreatic cancer cells under normal glucose conditions. Hyperglycaemia however, protected against the metformin-induced growth inhibition. The anti-proliferative actions of metformin were associated with an activation of AMP-activated protein kinase AMPK^Thr172^ together with an inhibition of the insulin/insulin-like growth factor-I (IGF-I) receptor activation and downstream signalling mediators IRS-1 and phosphorylated Akt. Furthermore, exposure to metformin during normal glucose conditions led to increased apoptosis as measured by poly(ADP-ribose) polymerase (PARP) cleavage. In contrast, exposure to high glucose levels promoted a more robust IGF-I response and Akt activation which correlated to stimulated AMPK^Ser485^ phosphorylation and impaired AMPK^Thr172^ phosphorylation, resulting in reduced anti-proliferative and apoptotic effects by metformin.

**Conclusion:**

Our results indicate that metformin has direct anti-tumour activities in pancreatic cancer cells involving AMPK^Thr172^ activation and suppression of the insulin/IGF signalling pathways. However, hyperglycaemic conditions enhance the insulin/IGF-I responses resulting in an altered AMPK activation profile and prevent metformin from fully switching off the growth promoting signals in pancreatic cancer cells.

## Background

Over recent decades the incidence of metabolic disorders, such as obesity and type 2 diabetes mellitus, has increased as a consequence of westernized lifestyle and changes in diet. These conditions are in turn associated with an increased risk of developing cancer [1-3]. Epidemiological studies have demonstrated that obesity and type 2 diabetes are among the top three modifiable risk factors for pancreatic cancer [2,4-8]. Almost 80% of pancreatic cancer patients present with either new-onset type 2 diabetes or impaired glucose tolerance at the time of diagnosis [9,10]. The relationship between type 2 diabetes and pancreatic cancer is complex and it remains unclear whether type 2 diabetes contributes to the development of pancreatic cancer or if precancerous cells cause the diabetes. Individuals with elevated fasting glucose and glycated haemoglobin (H_b_A_1c_) levels [11,12], or with higher c-peptide or insulin levels have a two to four-fold increase in the risk of pancreatic cancer [1,7]. Type 2 diabetes patients also demonstrate an increased risk of pancreatic cancer-related death as compared with those without diabetes [13]. Type 2 diabetes is characterized by hyperglycaemia and peripheral insulin resistance with compensatory hyperinsulinemia. Aside from its metabolic actions, insulin can mediate direct mitogenic effects through the insulin receptor (IR) and insulin-like growth factor I (IGF-I) receptor (IGF-IR). Insulin may also affect the cancer risk indirectly *via* increased production and bioavailability of IGF-I [6,14]. Additionally, hyperglycaemia can increase the sensitivity to IGF-I [4], thereby enhancing its mitogenic potential and providing an additional link between type 2 diabetes and cancer.

Insulin-sensitizing and glucose lowering drugs, such as metformin, are used as first-line treatment in the management of type 2 diabetes to improve glycaemic control in patients with insulin resistance. The key metabolic action of metformin involves the inhibition of hepatic glucose secretion, which consequently decreases the hyperinsulinemia. This mechanism is mediated *via* activation of the energy-sensing AMP-activated protein kinase (AMPK) in hepatocytes, through the liver kinase B1 (LKB1) signalling pathway [15]. Although metformin can lower blood glucose, the levels rarely remain within the normal range and as the type 2 diabetes progresses, additional medication such as exogenous insulin is often required to control patients’ hyperglycaemia [16,17]. In addition to its anti-diabetic effects, metformin has recently been postulated to have a protective role against cancer. Epidemiological and retrospective studies have demonstrated that diabetic patients taking metformin not only have a lower incidence of pancreatic cancer, but also an improved cancer outcome [18-21]. The indicated anti-neoplastic activity of metformin may relate to reduced plasma insulin concentrations or by direct effects on the tumour cells. Recent studies suggest that metformin-induced AMPK activation at Thr^172^ inhibits the central growth control node mammalian target of rapamycin mTOR, thus preventing protein synthesis and cell proliferation [22]. Metformin has recently been shown to possess anti-tumour effects, both in AMPK-dependent and independent manners [23-25].

Although an increasing number of studies demonstrate the anti-tumour effects of metformin, relatively little is known about the effects and underlying mechanisms of metformin on pancreatic cancer cells. The goal of this study was to examine the direct effects of metformin on human pancreatic cancer cells in the context of normal or elevated glucose levels. Effects on proliferation, apoptosis, AMPK activation and influence on and by the IGF-I pathway were analysed.

## Methods

### Materials

All chemicals and reagents were purchased from Sigma Aldrich (St. Louis, Mo, USA) unless stated otherwise. Cell culture media, penicillin/streptomycin and fetal bovine serum (FBS) were purchased from Invitrogen (Paisley, UK). IGF-I was purchased from GroPep (Adelaide, Australia). MTT; Cell Proliferation Kit I was derived from Roche (Mannheim, Germany). Anti-cleaved PARP, anti-phospho-AMPK^Thr172^, anti-phospho-AMPK^Ser485^, anti-AMPK, anti-IRS-1, anti-phospho-IGF-IRβ/phospho-IRβ, anti-phospho-Akt^Ser473^ and anti-Akt antibodies were purchased from Cell Signaling Technology Inc. (Beverly, MA, USA). Anti-IGF-IRβ was obtained from Santa Cruz Biotechnology (Santa Cruz, CA, USA) and anti-GAPDH from Millipore (Temecula, CA, USA).

### Cell culture

The human pancreatic adenocarcinoma cell lines AsPC-1, BxPC-3, PANC-1 and MIAPaCa-2 were purchased from ATCC-LGC Standards (Manassas, VA, USA). The cells were maintained in RPMI1640 or DMEM supplemented with 10% FBS and antibiotics (100 U/ml penicillin and 100 μg/ml streptomycin) in a humified 5% CO_2_ atmosphere at 37°C. All experiments were performed in glucose-free RPMI1640 or DMEM supplemented with 5 mM (normal) or 25 mM (high) D-glucose, 2 mM L-glutamine and antibiotics as above (serum-free media; SFM), unless stated otherwise.

### MTT proliferation assay

Cells were plated (10 × 10^3^ cells/well) in 96-well plates in growth media with 5 mM glucose for 24 h before switching to SFM with 5 mM or 25 mM glucose for another 24 h. Cells were subsequently dosed with increasing concentrations of metformin (0–20 mM) in SFM with 5 mM or 25 mM glucose in sextuplicates (n = 6 wells). SFM with either 5 mM or 25 mM was used as control. Following incubation for 24–72 h, cell proliferation was assessed by MTT according to the manufacturer’s instructions. The samples were measured on a Labsystems Multiskan Plus plate reader (test wavelength 595 nm, reference wavelength 660 nm) using the DeltaSoft JV software (BioMetallics Inc., Princeton, NJ, USA).

### Western immunoblotting

Cells were cultured (6 × 10^5^ cells/well) in 6-well plates for 24 h. After an additional 24 h in normal glucose SFM, the cells were dosed with metformin (0–20 mM) in SFM or 1% FBS SFM with 5 or 25 mM glucose for 24 h. Cells were then spiked with IGF-I (100 ng/ml) as indicated for the final 15 min of incubation. Cells were lysed as previously described [26]. Protein concentrations were determined using BCA protein assay reagent kit (ThermoFisherScientific, Waltham, MA, USA). Lysates were dissolved in Laemmli buffer, boiled for 5 minutes and separated (60–65 μg protein per lane) by SDS-PAGE (8% or 12%) and transferred to 0.2 μm Hybond-C extra nitrocellulose membrane (Amersham Biosciences, Buckinghamshire, UK). The membranes were blocked with 5% (w/v) milk in Tris-buffered saline Tween-20 (TBST) and probed overnight (4°C) with the indicated antibodies, all used at dilutions of 1:1000. Immunoblotted proteins were detected using HRP-conjugated secondary antibodies and visualized by SuperSignal West Extended Duration Substrate (ThermoFisherScientific) using BioRad Chemidoc XRS + system and Image lab software.

### Statistical analysis

Proliferation data are expressed as means ± SE of six replicate wells. Densitometry analyses of Western blot data were performed using Image J software (NIH, USA) and are expressed as means ± SE of three individual experiments, unless stated otherwise. Statistical analyses were performed by one- or two-way ANOVA with Bonferroni post hoc test using GraphPad prism software. A *P*-value of <0.05 was considered statistically significant.

## Results

### Metformin acts as a growth inhibitor for human pancreatic cancer cells

**Figure 1:**
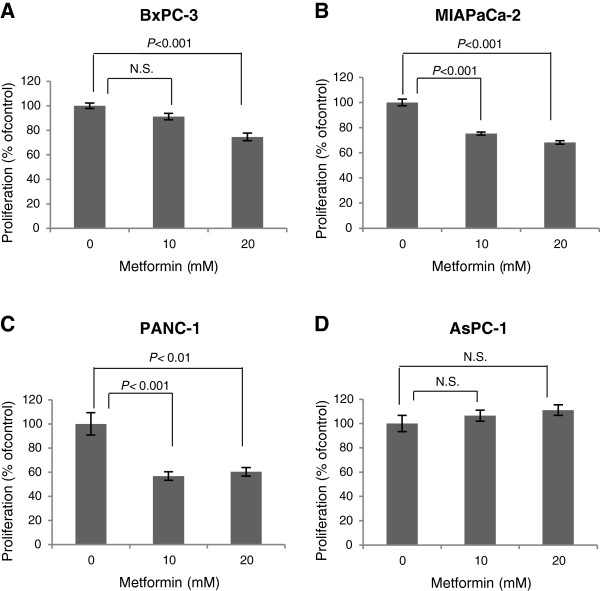
**Metformin acts as a growth inhibitor for human pancreatic cancer cells.** (**A**) BxPC-3, (**B**) MIAPaCa-2, (**C**) PANC-1 and (**D**) AsPC-1 pancreatic cancer cells were seeded in 96-well plates in normal (5 mM) glucose-containing growth media with 10% FBS for 24h, followed by 24 h in 5 mM glucose-containing SFM. The cells were then dosed with or without metformin in normal glucose SFM for an additional 24 h. Results are shown as percentage of SFM control and represent mean ± SE of six replicate wells. *P*-values are based on one-way ANOVA with Bonferroni post hoc test. N.S., not significant. One representative out of three independent experiments is shown.

To examine the effect of metformin on cell proliferation, a panel of human pancreatic cancer cell lines were exposed to metformin for 24 h in normal glucose levels (5 mM). Metformin induced a significant 20-40% growth inhibition of BxPC-3 (*P* < 0.001; Figure 1A), MIAPaCa-2 (*P* < 0.001; Figure 1B) and PANC-1 (*P* < 0.01; Figure 1C), whereas no growth inhibitory effect was observed on AsPC-1 pancreatic cancer cells (Figure 1D).

### Hyperglycaemia suppresses metformin-induced growth inhibition

**Figure 2:**
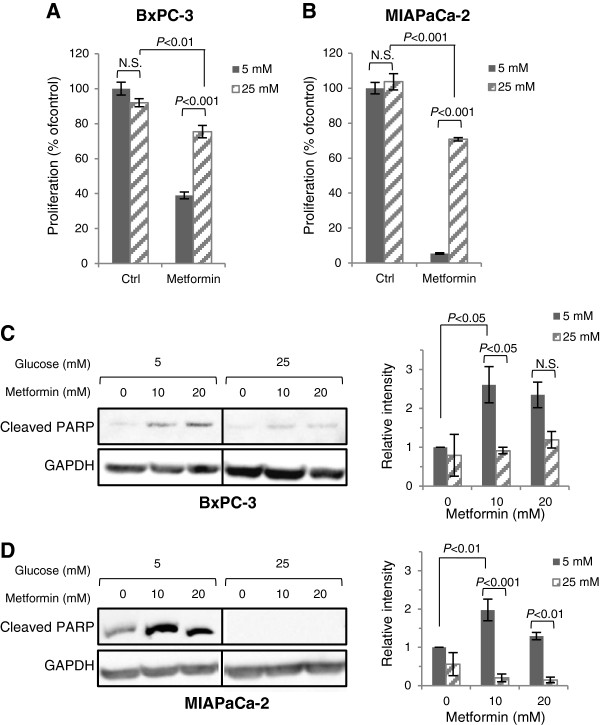
**High glucose reduces metformin-induced growth suppression and apoptosis.** (**A**) BxPC-3 and (**B**) MIAPaCa-2 cells were treated with or without 10 mM metformin in normal (5 mM) or high (25 mM) glucose-containing SFM for 72 h and proliferation was assessed by MTT assay. Graphs represent changes in proliferation and mean ± SE of six replicate wells are depicted. *P* < 0.05, two-way ANOVA with Bonferroni post hoc test. N.S., not significant. (**C**) BxPC-3 and (**D**) MIAPaCa-2 cells were treated in 1% FBS SFM with either 5 or 25 mM glucose, with or without metformin for 24 h and processed for levels of cleaved PARP as an indicator of apoptosis by Western immunoblotting. Histograms show densitometry evaluation of relative difference in cleaved PARP intensity and represent means ± SE of three independent experiments. *P*-values are based on two-way ANOVA with Bonferroni post hoc test. Blots shown are representative assays from three independent experiments.

The influence of increased levels of glucose on the sensitivity to metformin was examined. When BxPC-3 and MIAPaCa-2 cells were exposed to metformin for 72 h under hyperglycaemic conditions (25 mM), the proliferation was decreased by 18% and 32%, respectively. Hyperglycaemia significantly reduced the efficacy of metformin as compared with normal (5 mM) glucose levels where a 56% and 95% growth inhibition was obtained in BxPC-3 and MIAPaCa-2, respectively (*P* < 0.001; Figure 2A, B). The growth inhibitory effects by metformin in normal glucose conditions correlated with a significant induction of cleaved PARP, as an indicator of apoptosis (*P* < 0.05; Figure 2C, D). In contrast, levels of cleaved PARP in response to metformin were significantly decreased or absent at high glucose conditions, consistent with the reduced sensitivity of BxPC-3 and MIAPaCa-2 to metformin.

### Hyperglycaemia impairs pAMPK^Thr172^, but not pAMPK^Ser485^ activation

**Figure 3:**
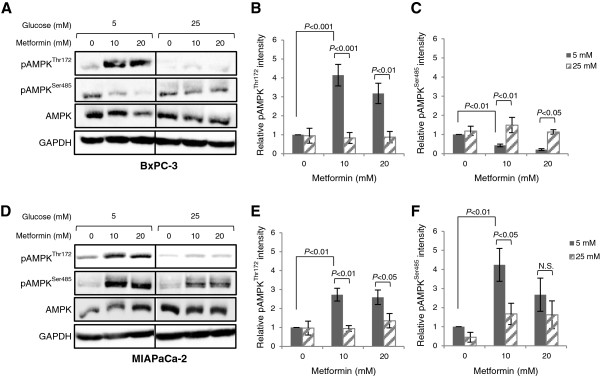
**Hyperglycaemia impairs pAMPK**^**Thr172**^**, but not pAMPK**^**Ser485 **^**activation.** (**A**-**C**) BxPC-3 and (**D**-**F**) MIAPaCa-2 cells were treated with or without metformin in normal (5 mM) or high (25 mM) glucose-containing media with 1% FBS for 24 h and processed for immunoblot analysis using antibodies against pAMPK^Thr172^, pAMPK^Ser485^ and AMPK. GAPDH is shown as a loading control. Graphs display densitometry measurements of relative levels of AMPK protein phosphorylation at 5 mM (black bars) or 25 mM (striped bars) glucose conditions and represent means ± SE of three independent experiments. *P*-values are based on two-way ANOVA with Bonferroni post hoc test. N.S. not significant. One representative blot from three independent experiments is shown.

To determine if the reduced efficacy of metformin during hyperglycaemic conditions was related to altered AMPK activation, metformin-stimulated AMPK phosphorylation in BxPC-3 and MIAPaCa-2 cells during normal or high glucose conditions was examined. These results show that metformin stimulated AMPK^Thr172^ phosphorylation in cells cultured in normal glucose (*P* < 0.01; Figure 3A, B, D, E). In BxPC-3 cells, metformin-induced AMPK^Thr172^ phosphorylation corresponded to a decrease in basal AMPK^Ser485^ phosphorylation (Figure 3A-C). In contrast, exposure to hyperglycaemic conditions inhibited both basal and metformin-stimulated pAMPK^Thr172^ increase, whereas AMPK^S485^ phosphorylation remained stable. In MIAPaCa-2, metformin induced both AMPK^Thr172^ and AMPK^Ser485^ phosphorylation in 5 mM glucose (*P* < 0.01; Figure 3D-F). However, exposure to 25 mM glucose almost completely inhibited the metformin-induced increase in AMPK^Thr172^ phosphorylation, while AMPK^Ser485^ phosphorylation was still present, similar to the effects observed in BxPC-3.

### Metformin modulates IRS-1 levels and Akt phosphorylation

**Figure 4:**
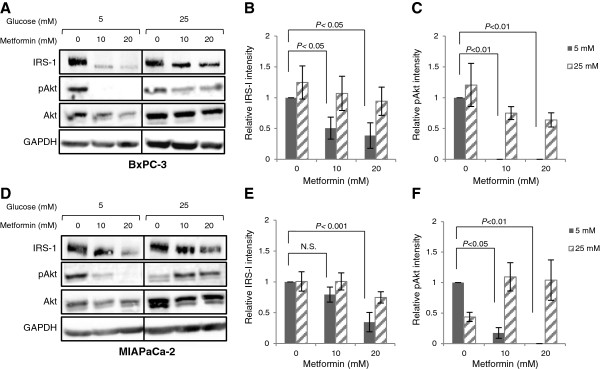
**Metformin modulates IRS-1 levels and Akt phosphorylation.** (**A**-**C**) BxPC-3 and (**D**-**F**) MIAPaCa-2 cells were treated in 1% FBS SFM with either normal (5 mM) or high (25 mM) glucose, with or without metformin for 24 h and processed for immunoblot analysis using antibodies against IRS-1, pAkt and Akt. GAPDH is shown as a loading control. One representative blot is shown. Graphs display densitometry measurements of relative levels of IRS-1 and pAkt at 5 mM (black bars) or 25 mM (striped bars) glucose conditions and represent means ± SE of three independent experiments. *P*-values are based on two-way ANOVA with Bonferroni post hoc test. N.S. not significant.

Having shown that high levels of glucose altered the responsiveness to metformin and influenced the AMPK activation pattern, we then examined the involvement of the insulin/IGF-I signalling pathways. As shown in Figure 4, metformin exposure resulted in a significant decrease in basal levels of IRS-1 (*P* < 0.05) and Akt^Ser473^ (*P* < 0.01) phosphorylation in a dose-dependent manner during normal glucose conditions. In contrast, a high glucose environment counteracted the metformin-mediated IRS-1 and pAkt suppression in BxPC-3 (Figure 4A) and MIAPaCa-2 (Figure 4B).

### IGF-I and hyperglycaemia enhance AMPK^Ser485^ phosphorylation

**Figure 5:**
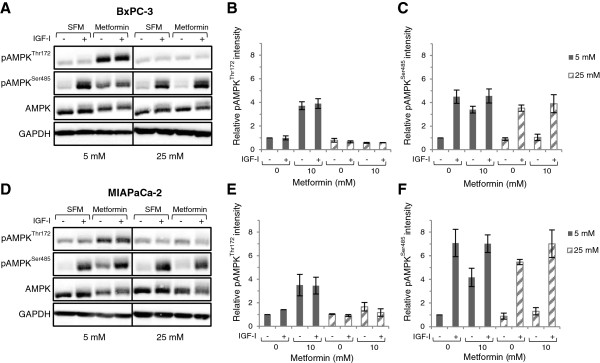
**IGF-I and hyperglycaemia enhance pAMPK**^**Ser485 **^**phosphorylation.** (**A**-**C**) BxPC-3 and (**D**-**F**) MIAPaCa-2 cells were treated in normal (5 mM) or high (25 mM) glucose-containing SFM, with or without 10 mM metformin for 24 h. Cells were spiked with IGF-I (100 ng/ml) as indicated for the final 15 min prior to being lysed and processed for pAMPK^Thr172^, pAMPK^Ser485^ and AMPK abundance by Western immunoblotting. GAPDH is shown as loading control. One representative blot is shown. Graphs display densitometry measurements of relative levels at 5 mM (black bars) or 25 mM (striped bars) glucose conditions and represent means ± SE of two (**B**) or three (**A**, **C**-**D**) independent experiments.

Having found that metformin influenced the IGF-I signalling mediators under normal glucose levels, we next addressed the influence by IGF-I on AMPK activation. No changes in the basal or metformin-induced phosphorylation of AMPK^Thr172^ were observed in response to IGF-I stimulation at normal glucose (Figure 5A-B, D-E). Instead, stimulation of BxPC-3 (Figure 5A, C) and MIAPaCa-2 (Figure 5D, F) cells with 100 ng/ml IGF-I caused a strong phosphorylation of AMPK^Ser485^. In hyperglycaemia, the IGF-I-induced AMPK^Ser485^ phosphorylation was sustained in both cell lines. This suggested that exposure to IGF-I in combination with higher glucose concentrations stimulated AMPK^Ser485^ phosphorylation, which shifted the AMPK^Thr172^ to AMPK^Ser485^ balance.

### Metformin inhibits IGF-I-stimulated IGF-IR and Akt activation

**Figure 6:**
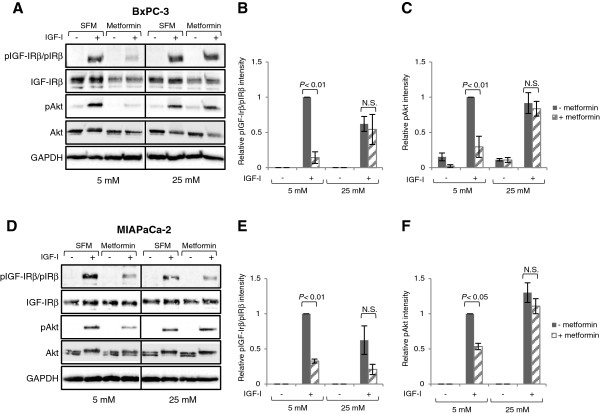
**Metformin inhibits IGF-I-stimulated IGF-IR and Akt activation in normal glucose.** (**A**-**C**) BxPC-3 and (**D**-**F**) MIAPaCa-2 cells were treated in normal (5 mM) or high (25 mM) glucose-containing SFM, with or without 10 mM metformin for 24 h. Cells were spiked with IGF-I (100 ng/ml) as indicated for the final 15 min prior to lysis. pIGF-IRβ/pIRβ, IGF-IRβ, pAkt, and Akt were assessed by Western immunoblotting. GAPDH is shown as loading control. One representative blot is shown. Graphs display densitometry measurements of relative levels at 5 mM (black bars) or 25 mM (striped bars) glucose conditions and represent means ± SE of three independent experiments. *P*-values are based on two-way ANOVA with Bonferroni post hoc test. N.S. not significant.

After studying the influence by IGF-I on AMPK activation, we examined modulation of the IGF-IR pathway by metformin. Interestingly, metformin potently inhibited the activating phosphorylation of the IGF-IRβ/IRβ (*P* < 0.01), and subsequently also the downstream phosphorylation of Akt in both BxPC-3 (*P* < 0.01) and MIAPaCa-2 (*P* < 0.05) cells at normal glucose levels (Figure 6). However, in the hyperglycaemic situation, the IGF-I-mediated IGF-IRβ/IRβ and Akt activation appeared to be more robust and could override the inhibitory action of metformin. The sustained IGF-IRβ/IRβ and Akt activation correlated with the observed activation of AMPK^Ser485^, supporting the hypothesis of a link between the two pathways.

## Discussion

Type 2 diabetes or impaired glucose tolerance frequently occurs in pancreatic cancer patients. Compared to other treatments, diabetic patients on metformin have a reduced risk of approximately 40% of developing several types of cancer, including pancreatic cancer [19-21]. However, the molecular relationships underlying the metabolic and suggested anti-cancer actions of metformin remain poorly understood. Additionally, the importance of optimal glucose control for the anti-tumour effects of metformin has not been fully established. In this study, we describe direct anti-proliferative actions by metformin using *in vitro* models of pancreatic cancer. In addition, we demonstrate that elevated glucose levels impair AMPK activation and reduce the efficacy of metformin. Importantly, we show a novel role for metformin on human pancreatic cancer cells that may contribute to its indicated anti-cancer actions among type 2 diabetic patients.

Metformin is believed to act mainly through activation of the energy-conserving LKB1-AMPK pathway. Physiological activation of the AMPK metabolic checkpoint in response to nutrient depletion and energy stress suppresses energy-consuming cellular processes such as protein synthesis and cell division. We found that metformin during normal glucose conditions significantly reduced proliferation and promoted apoptosis through PARP cleavage in pancreatic cancer cells with functional LKB1 (MIAPaCa-2, BxPC-3 and PANC-1), while being incapable of suppressing growth under the same conditions in AsPC-1 pancreatic cancer cells. AsPC-1 cells have previously been reported to carry an epigenetic inactivation of LKB1 [27]. Our findings are consistent with prior observations, showing pro-apoptotic actions on breast cancer cells [28,29] and that a functional LKB1 was required for the *in vitro* anti-proliferative effect of metformin [25,30].

Previous work indicates that metformin functions by activating AMPK at Thr^172^ with subsequent downstream inhibition of the growth promoting PI3K/Akt/mTOR pathway [29,31,32]. Similarly, we also found the growth inhibitory properties of metformin to be associated with the activation of AMPK^Thr172^ in pancreatic cancer cells. Under hyperglycaemic conditions, the efficacy of metformin was reduced with less anti-proliferative and pro-apoptotic activity observed. Other investigators have reported that lung and colon carcinoma cells were more sensitive to metformin-induced growth inhibition at low glucose concentrations, while no significant effect of metformin on cell death was observed in high glucose conditions [30]. Similarly, a recent study demonstrated anti-proliferative effects on pancreatic cancer cells by metformin at the low 0.05-1 mM range at normal (5 mM) glucose conditions [33]. This study is in concordance with our data demonstrating direct anti-tumour effects of metformin and supports our findings of enhanced sensitivity at physiological normal glucose levels. We have now shown that the lower anti-proliferative effect of metformin on pancreatic cancer cells at higher glucose levels correlates to an impaired AMPK^Thr172^ activation and a shifted balance from AMPK^Thr172^ towards AMPK^Ser485^ activation. The role of AMPK^Ser485^ in the complex AMPK signaling network is at present not completely clear and conflicting reports exist. A recent study indicated that endogenous protein kinase A (PKA)-induced activation of AMPK^Ser485^ in pancreatic beta cells did not affect the phosphorylation status of AMPK^Thr172^. However, the activation of Thr^172^ and Ser^485^ were inversely correlated in response to glucose [34]. Other studies have proposed that PKB/Akt-induced phosphorylation of AMPK^Ser485^ can counteract AMPK^Thr172^ activation, thereby reducing the effects of metformin [31,32,35].

Hyperinsulinemia with resulting increased circulating levels of IGF-I have been suggested to play a role in the connection between type 2 diabetes and cancer [36]. Activation of the IR and IGF-IR result in receptor autophosphorylation and recruitment of insulin receptor substrate (IRS) 1, which in turn activates the PI3K/Akt pathway leading to protein synthesis and cell survival [14]. The responsiveness to IGF-I can be enhanced by exposure to high glucose concentrations [4], which may then further promote cancer progression. In pancreatic cancer cells, IGF-I stimulated a pronounced phosphorylation of Akt and also AMPK^Ser485^. However, at physiologically normal glucose levels, IGF-I stimulated AMPK^Ser485^ phosphorylation did not appear to antagonize pharmacological activation of AMPK^Thr172^ by metformin. Instead, we established that metformin under these conditions suppressed IGF-IR/IR phosphorylation causing a downstream inhibition of both basal and IGF-I stimulated Akt phosphorylation. It is well established that IGF-IR *via* activation by its ligands transmits mitogenic signals leading to the survival and proliferation of multiple types of cancer. Mechanisms by which metformin inhibits these pathways may thus contribute to the anti-tumour effects previously observed in response to metformin. Studies in other cell types have shown that during normal glucose conditions, AMPK^Thr172^ can phosphorylate inhibitory serine residues on IRS-1, which prevents signalling through the PI3K/Akt pathway [29,37]. However, studies have also shown that Akt at high glucose conditions can inhibit AMPK by phosphorylation of Ser^485^, which prevents activation of Thr^172^ and thereby the action of metformin [29,31,35,37,38]. In keeping with this, we observed a strong activation of Akt and AMPK^Ser485^ following IGF-I stimulation at high glucose, which was sustained after exposure to metformin. At high glucose, IGF-I induced Akt and AMPK^Ser485^ phosphorylation appeared to correlate with a further reduction of the already-impaired AMPK^Thr172^ phosphorylation by metformin.

## Conclusions

**Figure 7:**
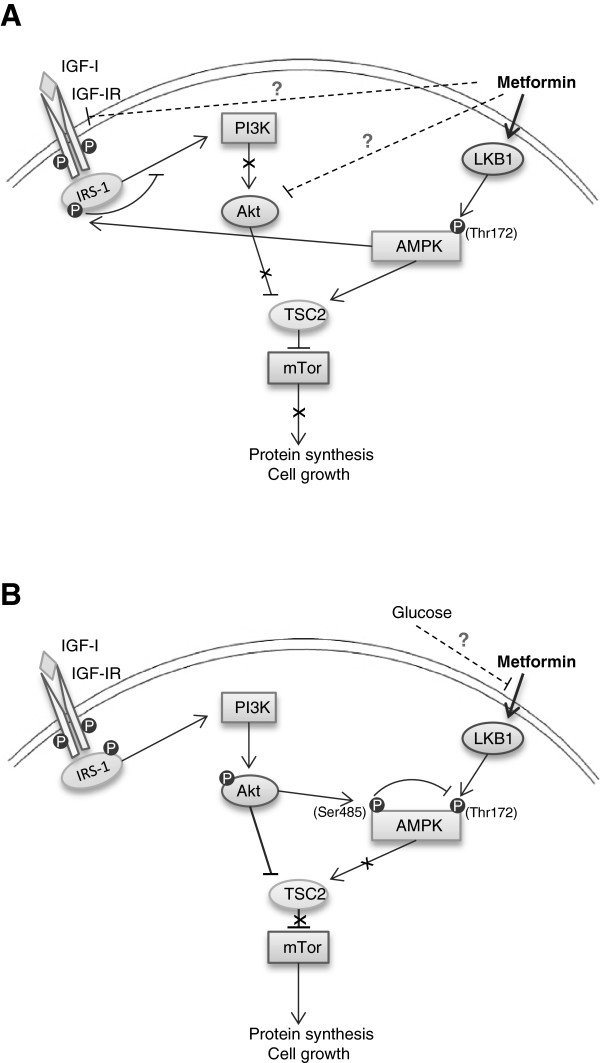
**Overview of selected AMPK and IGF-IR signalling pathways relevant to this study in normal (A) and hyperglycaemic (B) conditions.** In normal conditions, metformin activates AMPK^Thr172^ through the LKB1 signalling pathway. Activated AMPK inhibits cell growth by repressing mTOR activity through activation of TSC2 and phosphorylation of inhibitory serine residues on IRS-1. In addition, as demonstrated in the present study, metformin suppresses IGF-IR activation and reduces IRS-1 levels, which disable further signalling through the PI3K/Akt pathway. During hyperglycaemia, signalling through the IGF-IR pathway appears more robust, leading to phosphorylation of Akt and AMPK^Ser485^. This inhibitory Ser485 site can inhibit activation of AMPK^Thr172^, possibly contributing to the impaired growth inhibitory effect by metformin at high glucose conditions.

The findings of the current study using human pancreatic cancer cells add novel information to the indications of direct anti-tumour actions by metformin on transformed epithelial cells. Metformin mediated its effects through activation of AMPK^Thr172^ together with inhibition of the IR/IGF-IR signalling pathway (Figure 7). Hyperglycaemia, with and without IGF-I, reduced the sensitivity to metformin and counteracted the growth inhibitory effect otherwise exerted by the drug. Our data suggests that metformin may have beneficial effects on tumour prevention or protection in non-diabetic patients with normal glucose levels. Importantly, these data indicate that optimizing glucose control in type 2 diabetic patients may improve the beneficial anti-tumour actions provided by metformin and should thus be further investigated. Due to the strong associations between type 2 diabetes and pancreatic cancer, evaluating the potential beneficial effects by metformin, as well as the impact by different glucose levels, should be considered of utmost importance. Increased understanding of the relationship between the two conditions may improve both future treatment strategies as well as potentially providing possibilities of novel early diagnostic approaches.

## Abbreviations

AMPK: Adenosine monophosphate activated protein kinase; IGF: Insulin-like growth factor; IGF-IR: Insulin-like growth factor type I receptor; IR: Insulin receptor; IRS-1: Insulin receptor substrate-1; PARP: Poly(ADP-ribose) polymerase.

## Competing interests

The authors declare that they have no competing interests.

## Authors’ contributions

AHR designed the study. EK, KS and AHR made significant contributions to the experimental design, acquisition and interpretation of data, manuscript preparation and editing. RA and AHR provided funding for the study and manuscript editing. All authors read and approved the final manuscript.

## Pre-publication history

The pre-publication history for this paper can be accessed here:

http://www.biomedcentral.com/1471-2407/13/235/prepub
